# The Effects of Obesity-Related Anthropometric Factors on Cardiovascular Risks of Homeless Adults in Taiwan

**DOI:** 10.3390/ijerph17186833

**Published:** 2020-09-18

**Authors:** Ching-Lin Chen, Mingchih Chen, Chih-Kuang Liu

**Affiliations:** 1 Graduate Institute of Business Administration, Fu Jen Catholic University, New Taipei City 242, Taiwan; a1554@tpech.gov.tw; 2 Taipei City Hospital, Taipei City 10341, Taiwan; 3 Artificial Intelligence Development Center, Fu Jen Catholic University, New Taipei City 242, Taiwan; 4 Department of Urology, Fu-Jen Catholic University Hospital, New Taipei City 242, Taiwan

**Keywords:** homeless adults, BMI, WC, WHR, WHtR, cardiovascular risk

## Abstract

Homelessness is a pre-existing phenomenon in society and an important public health issue that national policy strives to solve. Cardiovascular disease (CVD) is an important health problem of the homeless. This cross-sectional study explored the effects of four obesity-related anthropometric factors—body mass index (BMI), waist circumference (WC), waist-to-hip ratio (WHR), and waist-to-height ratio (WHtR)—on cardiovascular disease risks (expressed by three CVD markers: hypertension, hyperglycemia, and hyperlipidemia) among homeless adults in Taipei and compared the relevant results with ordinary adults in Taiwan. The research team sampled homeless adults over the age of 20 in Taipei City in 2018 and collected 297 participants. Through anthropometric measurements, blood pressure measurements, and blood tests, we calculated the obesity-related indicators of the participants and found those at risks of cardiovascular disease. The results showed that the prevalence of hypertension, hyperglycemia, and hyperlipidemia in homeless adults was significantly higher than that of ordinary adults in Taiwan. Among the four obesity-related indicators, WHtR showed the strongest association with the prevalence of hypertension and hyperlipidemia, followed by WHR, both of which showed stronger association than traditional WC and BMI indicators. It can be inferred that abdominal obesity characterized by WHtR is a key risk factor for hypertension and hyperlipidemia in homeless adults in Taiwan. We hope that the results will provide medical clinical references and effectively warn of cardiovascular disease risks for the homeless in Taiwan.

## 1. Introduction

Homeless individuals are defined as people without a fixed residence and fixed work [1]. The last time a global survey was attempted—by the United Nations in 2005—an estimated 100 million people were homeless worldwide [2]. Homelessness is usually caused by health problems and social factors and is related to insufficient food, alcoholism, and lack of personal hygiene [3,4]. It can also easily lead to various diseases including mental illness, respiratory disease, cardiovascular disease, and various infectious diseases, as well as the inability to pay for medical expenses [5]. These complex medical and social issues have resulted in low life expectancy and high all-cause mortality among homeless people [6,7,8]. Medical care of the homeless is always an important public health issue.

Cardiovascular disease (CVD) is the most important health problem for the homeless and is a major cause of death among homeless adults, at rates that exceed those in non-homeless individuals [9,10,11]. Slockers et al. [12] found that CVD was the most important cause of death due to disease among the homeless, accounting for 22% of all-cause mortality, second only to unnatural accidental death (26%). Scott et al. [13] found that, among the homeless, the prevalence of diabetes, pre-diabetes, and metabolic syndrome (MetS) were at higher risk than in the general population. Szerlip and Szerlip [14] investigated the homeless in New Orleans and found that hypertension was present in 65% of the homeless but only in 52% of the non-homeless, and there was no difference in the prevalence of diabetes or total cholesterol. Lee et al. [15] found that, in North America, hypertension, high cholesterol, and diabetes were not more prevalent in the homeless than in the general population but were often poorly controlled. It was not easy to control hypertension, hyperlipidemia, and hyperglycemia for the homeless [16]. The prevalence of CVD risk for the homeless is often different in different regions, races, and countries.

Obesity can increase cardiovascular disease morbidity and mortality directly and indirectly. The direct effects are mediated by obesity-induced structural and functional adaptations of the cardiovascular system to accommodate excess body weight, as well as by adipokine effects on inflammation and vascular homeostasis [17]. There have been few studies on the impact of obesity factors on CVD risk of the homeless. For the general public, obesity can cause coronary heart disease, heart failure, stroke, atrial fibrillation, and sudden cardiac death and is a significant risk factor for hypertension, hyperglycemia, and hyperlipidemia [18,19,20]. Susceptibility to obesity-related cardiometabolic (CM) complications is not solely mediated by overall body fat mass but is largely dependent upon individual differences in regional body fat distribution and the ability of subcutaneous adipose tissue to expand [21]. Anthropometric indicators of overall obesity and abdominal obesity were often included in studies and compared to illustrate the responses to cardiovascular risks [22,23,24]. Amirabdollahian and Haghighatdoost [25] assessed the heart health of young people and found that waist circumference (WC) and waist-to-height ratio (WHtR) had stronger association with CM risk compared with body-weight-related measures. Nyamdorj et al. [26] studied data from 16 participating cohorts in seven countries (Chinese, Filipino, native Japanese and Japanese living in Brazil, Mongolian, native Asian Indian living in India, and migrant Asian Indian living in Mauritius) through collaborative analysis and found that WHtR had the strongest association with diabetes while body mass index (BMI) had the strongest association with hypertension in Asians.

The current study explored the effects of the obesity-related anthropometric factors on the risk of cardiovascular disease among homeless adults in Taipei City and tried to provide the government with recommendations to manage health policies for homeless adults. Statistics as of June 2018 showed that, in the past year, the public and private sectors in Taiwan provided 140,067 person-times of assistance to homeless people, including 2385 person-times of medical assistance [27]. In Taiwan, homeless adults had complex health problems, and health problems accounted for 57% of all the problems they had [28]. The government needs to provide effective healthcare services. This investigation was led by the Taipei City Hospital in Taiwan. The Taipei City Hospital and the Taipei City Social Bureau are the main institutions that take care of their health problems [29]. According to statistics from the Ministry of Health and Welfare of Taiwan, in 2018, the number of homeless people in Taiwan was 2603, of which the male-to-female ratio was 89.1%:10.9%. The number of homeless people in Taipei City was 669, and the male-to-female ratio was 85.3%:14.7%. Taipei was the city with the largest number of homeless people in Taiwan, accounting for 25.7% of the Taiwanese homeless population [28]. Research on healthcare issues of homeless people is of great significance to inclusive social welfare.

## 2. Materials and Methods

### 2.1. Study Design and Participants

The investigation was led by the Taipei City Hospital in Taiwan. In this cross-sectional study, the sample collection was targeted at the homeless adults in the Taipei City Homeless Counseling Organizations in 2018. The study collaborated with these organizations to carry out purposive sampling. The number of participants was 297, accounting for 44.4% of the Taipei city’s homeless population. The sample male-to-female ratio was 77.4%:22.6%, which was similar to Taipei’s overall ratio and was sufficiently representative [28]. Our sampled population was similar to the actual street sample.

The investigation process obtained participants’ anthropometric data (body height, body weight, waist circumference, hip circumference) and cardiovascular indicators (systolic blood pressure (SBP), diastolic blood pressure (DBP), fasting plasma glucose (FPG), total cholesterol (TC), triglyceride (TG), and high-density lipoprotein cholesterol (HDL-C)) through clinical examinations, and obtained participants’ demographic information (gender and age). The investigation period was from 1 April 2018–30 September 2018. This study was reviewed and approved by the Research Ethics Committee of Taipei City Hospital (Case No.: TCHIRB-10805021-E).

### 2.2. Testing Procedures

The anthropometric measurements included body height, body weight, waist circumference (WC), and hip circumference (HC). Body mass index (BMI), waist-to-hip ratio (WHR), and waist-to-height ratio (WHtR) were obtained by calculation. Cardiovascular indicator measurements included blood pressure measurement and blood sampling test. Blood glucose and lipid profile were collected by blood drawing after fasting for at least eight hours. The following testing procedures were conducted in accordance with the testing standards of the Ministry of Health and Welfare [30], and the team of nurses affiliated to the hospital was responsible for completing the testing.

#### 2.2.1. Anthropometric Measurement

Anthropometric measures included body height, body weight, waist circumference, and hip circumference. First, the participants removed their shoes and heavy clothes. Body height (cm) and body weight (kg) were recorded to the nearest 0.1 cm and 0.1 kg with an electronic height-weight scale. WC (cm) was measured to the nearest 0.1 cm with a flexible steel tape measure placed midway between the lowest rib and iliac crest when participants were in a standing position at the end of an exhalation. HC (cm) was measured to the nearest 0.1 cm at the widest part of the hip region in the standing position. Both circumferences were measured twice without difference over 2 cm, and the average was calculated as the result.

#### 2.2.2. Blood Pressure Measurement

During the measurement, the participant sat in a sitting position with moderate room temperature, stayed in a quiet and comfortable place, kept calm, wore loose-fitting clothes, and did not have tight sleeves. The measurement used a mercury sphygmomanometer to measure systolic (mmHg) and diastolic blood pressure (mmHg). After measuring the blood pressure three times, the average was calculated as the result, and the interval between each measurement was 10–15 min.

#### 2.2.3. Blood Test

Blood glucose and lipid profile were collected by blood drawing after fasting for at least 8 hours. During the measurement, the participant sat on a comfortable and safe blood collection chair with armrests. The nurse chose the appropriate needle and blood collection tube, wore gloves, selected the appropriate venous blood collection site, kept the needle stable during the collection, and avoided infection. Finally, fasting plasma glucose (mg/dl), total cholesterol (mg/dl), triglyceride (mg/dl), and high-density lipoprotein cholesterol (mg/dl) indicators were obtained through blood testing.

#### 2.2.4. Indicator Calculation and Classification

The calculations of obesity-related indicators were as follows: BMI = body weight (kg)/height squared (m^2^), WHR = WC (cm)/HC (cm), and WHtR = WC (cm)/height (cm).

According to the obesity standards for Taiwan residents formulated by the Ministry of Health and Welfare [31], the BMI classification was defined as overall obesity with a BMI value greater than 27 kg/m^2^; otherwise, it was non-obesity. The WC classification was defined as abdominal obesity if the WC value of men was greater than 90 cm or that of women was greater than 80 cm; otherwise, it was non-obesity. We adopted the WHR cutoff of obesity recommended by the Ministry of Health and Welfare in Taiwan [32,33], which was recommended as abdominal obesity if men’s WHR value was greater than 0.92 or women’s was greater than 0.88; otherwise, it was non-obesity. At present, there was no authoritative WHtR cutoff for obesity applicable to Taiwan residents. Based on the literature [33,34,35,36], we selected the WHtR obesity cutoff that was generally applicable: the WHtR classification was defined as abdominal obesity if the WHtR value was greater than 0.5; otherwise, it was non-obesity.

According to the diagnostic criteria of cardiovascular indicators defined by the Ministry of Health and Welfare [37], hypertension was defined as those with systolic blood pressure ≥140 mmHg or diastolic blood pressure ≥90 mmHg or receiving drug treatment for hypertension; hyperglycemia was defined as fasting plasma glucose ≥126 mg/dl, or receiving drug treatment for blood glucose; hyperlipidemia was defined as total cholesterol ≥240 mg/dl or triglycerides ≥200 mg/dl or taking hypolipidemic drugs. We used the above criteria to define cardiovascular disease makers and cardiovascular disease risks.

### 2.3. Statistical Analyses

We used R software of version 3.6.3 (R core team, Vienna, Austria) to assess the prevalence of cardiovascular disease markers and the proportion of obesity among homeless adults in Taipei and identified the main anthropometric factors for cardiovascular disease risks. Descriptive statistics was used to show the magnitude and distribution of continuous variables. T-test and chi-square test were performed to analyze gender interference. The adjusted odds ratio (OR) with 95% confidence interval (CI) was calculated using logistic regression to estimate the association of obesity-related anthropometric factors with cardiovascular disease risks and assess the strengths of the association of BMI, WC, WHR, and WHtR. Statistical results were statistically significant with *p* < 0.05 * (significant), *p* < 0.01 ** (highly significant), and *p* < 0.001 *** (very highly significant).

## 3. Results

A total of 297 participants participated in the investigation. Two hundred and eighty valid data samples were obtained after processing the missing values. Through clustering, we analyzed the extreme values of the sample and found that there were a set of statistical outliers of SBP and DBP (SBP: 158 mmHg; DBP: 167 mmHg) and five statistical outliers of TG (TG: 994 mg/dl, 1074 mg/dl, 1111 mg/dl, 1114 mg/dl, 1407 mg/dl). It had been verified that these extreme values were the true values of the actual investigation, so these values were retained. Table 1 presents the statistical description of age and measurement indicator variables.

Table 2 lists the results of *t*-test and chi-square analysis by gender. For continuous variables, there were significant differences in WHR (*p*-value = 0.0013), WHtR (*p*-value = 0.014), TC (*p*-value = 0.0014), and HDL-C (*p*-value < 0.001) between the male and female groups. For categorical variables, only the classification of abdominal obesity marked by WC had a significant difference between gender groups (*p*-value < 0.001). On the whole, gender differences rarely interfered with the differences in indicators between male and female homeless adults.

Table 3 shows the results of the logistic regression for cardiovascular disease markers for obesity-related anthropometric factors. The odds ratios (ORs) showed clear comparisons between obesity-related indicators. After adjustment for gender and age, the odds ratios and 95% confidence intervals (CIs) for hypertension for each standard deviation (SD) increase in BMI, WC, WHR, and WHtR were 1.62 (1.23–2.11), 1.85 (1.28–2.34), 1.72 (1.32–2.24), and 1.60 (1.27–2.16), respectively. By using cutoffs to classify the factors, after BMI, WC, WHR, and WHtR values changed from non-obesity to obesity, the adjusted ORs and 95% CIs for hypertension were 2.09 (1.18–3.77), 2.00 (1.22–3.31), 2.74 (1.59–4.81) and 5.38 (2.89–10.54). Only the BMI category indicator was associated with hyperglycemia, and the adjusted OR was 2.02 (1.12–3.64). Also after adjustment for gender and age, the ORs and 95% CIs for hyperlipidemia for each SD increase in BMI, WC, WHR, and WHtR were 1.69 (1.34–2.30), 1.64 (1.28–2.35), 1.84 (1.41–2.54), and 1.73 (1.37–2.33). By using cutoffs to classify the factors, after BMI, WC, WHR, and WHtR values changed from non-obesity to obesity, the adjusted ORs and 95% CIs for hyperlipidemia were 2.95 (1.67–5.25), 3.03 (1.77–5.31), 3.53 (1.89–6.98), and 5.18 (2.53–11.81), respectively.

Through logistic regression analysis, we found that the four obesity-related indicators were all risk factors of hypertension and hyperlipidemia. Among the category indicators that characterize obesity, WHtR had the strongest association with hypertension, followed by WHR, and the weakest was WC. For hyperlipidemia, WHtR still had the strongest association, followed by WHR, and the weakest was BMI.

## 4. Discussion

The present study explored the effects of the obesity-related anthropometric factors on the risk of cardiovascular disease among homeless adults in Taipei City. The results showed the four obesity-related indicators (BMI, WC, WHR, and WHtR) are all risk factors of hypertension and hyperlipidemia in homeless adults. The WHtR indicator showed the strongest association with the prevalence of hypertension and hyperlipidemia, followed by WHR, both of which showed stronger association than traditional BMI and WC indicators. But in particular, only the BMI category factor was associated with the prevalence of hyperglycemia in homeless adults. We found that the WHtR obesity classification with 0.5 as cutoff was very sensitive to the segmentation of patients with hypertension and hyperlipidemia, far exceeding the association between the other three obesity factors and the two CVD markers. Therefore, it can be inferred that abdominal obesity characterized by WHtR is an important risk factor for hypertension and hyperlipidemia in homeless adults in Taiwan.

According to the statistical yearbook of health promotion 2018 of the National Health Administration of the Ministry of Health and Welfare of Taiwan, among adults over 20 in Taiwan, the prevalence of hypertension was 25.7% (male: 29.3%; female: 22.3%), the prevalence of hyperglycemia was 9.3% (male: 10%; female: 8.7%), and the prevalence of hyperlipidemia was 21.6% (male: 24.5%; female: 18.8%) [38]. In our results, in homeless adults over 20, the prevalence of hypertension was 49% (male: 48%; female: 49%), the prevalence of hyperglycemia was 28% (male: 34%; female: 22%), and the prevalence of hyperlipidemia was 37% (male: 30%; female: 43%). According to the 2013–2017 National Nutrition and Health Survey in Taiwan (NAHSIT) data from the National Health Administration, among adults over 20 years old in Taiwan, the prevalence of abdominal obesity (by WC) was 42.5% (male: 38%; female: 46.6%), and the prevalence of overall obesity (by BMI) was 22.3% (male: 25.3%; female: 19.4%) [39]. We found that the abdominal obesity rate (by WC) was 61% (male: 48%; female: 73%), and the overall obesity rate (by BMI) was 25% (male: 24%; female: 27%) in homeless adults. Our study and the above statistics of the general population in Taiwan both used the same cutoff standards. We have also performed gender-weighted calculations based on Table 2 to make the comparisons more reasonable. We found that the prevalence of hypertension, hyperglycemia, and hyperlipidemia, and the proportion of abdominal obesity among homeless adults in Taiwan were significantly higher than those of ordinary Taiwanese residents.

The above comparisons showed that the risk of cardiovascular disease and the prevalence of obesity of the homeless in Taiwan both were higher than those of ordinary people. But the phenomenon might be different in other parts of the world. Studies in North America found that hypertension, high cholesterol, and diabetes were not more prevalent in the homeless than in the general population [14,15]. One study in Boston found that the weight distribution of the homeless was not statistically different from that of the general population [40]; another study of homeless people in 11 US cities showed that 57% of homeless adults were overweight or obese, which was less than the 68% of obese or overweight individuals in the general US population [41]. Therefore, homeless people in different countries and regions might have different cardiovascular health and obesity characteristics. However, the reasons for the environmental and human body mechanisms behind these regionally different phenomena remain to be explored.

So, on the one hand, we speculated that abdominal obesity (especially characterized by WHtR) was the main risk factor for hypertension and hyperlipidemia among homeless adults in Taiwan; on the other hand, the risk of cardiovascular disease among homeless people in Taiwan was indeed a serious crisis, at least compared to North America. The 2019 statistics of death causes in Taiwan showed that, among the top 10 causes of death, the second place was heart diseases, the fourth place was cerebrovascular diseases, the fifth place was diabetes, and the eighth place was hypertensive diseases [42]. Chronic cardiovascular diseases account for four of the top 10 causes of death. Therefore, the cardiovascular health crisis of Taiwanese homeless people will only be more severe than that of the general public.

This study has several advantages. First of all, this is the first systematic study for homeless adults in Taiwan. According to the literature, no other researchers in Taiwan has conducted relevant systematic data research and analysis on homeless adults. Second, the data of this research is very representative and precious. Homeless adults are highly dissociative and move around. Collecting data related to homeless adults is very difficult, and it is more necessary to consider their personal wishes before conducting investigations. It also has some limitations. First, we used cross-sectional research design, which is unable to prove causality. Second, we conducted preliminary health examinations on the study participants, and the investigation data excluded those who were unhealthy and unable to walk. Third, we categorized CV indicators based on abnormal standards, did not ask participants if they used medication control and treatment, so the prevalence might be slightly underestimated.

Our study hope to provide education concepts and improve the obesity factors of the homeless population, thereby reducing the rate of cardiovascular disease, reducing health care expenditure, and reducing the national society cost. We recommend that Taiwanese government agencies include more comprehensive inspection items in future surveys, such as the Questionnaire on the Cognitive Test of Drug Abuse Prevention or the Brief Symptom Rating Scale, the purpose of which is to be able to quickly understand the individual’s psychological care needs and then provide the required mental health services. Meanwhile, this study showed that WHtR had a stronger association with CVD risk than BMI, WC, and WHR for the homeless adults. Many studies suggested that WHtR is regarded as a better anthropometric indicator for evaluating central adiposity because of its independence of age, gender, and ethnicity [43]. We recommend that the Ministry of Health and Welfare of Taiwan refer to and draw up the WHtR cutoff standard suitable for Taiwan residents.

## 5. Conclusions

In summary, the results of this study showed that BMI, WC, WHR and WHtR were all risk factors of hypertension and hyperlipidemia in homeless adults in Taipei. The WHtR indicator showed the strongest association with the prevalence of hypertension and hyperlipidemia, followed by WHR, both of which showed stronger association than traditional BMI and WC indicators. It can be inferred that abdominal obesity characterized by WHtR is a key risk factor for hypertension and hyperlipidemia in homeless adults in Taiwan. This study is based on the actual anthropometric and blood sampling data of Taipei homeless adults. The data is representative, and the results can be extended to the Taiwan homeless adult.

## Figures and Tables

**Table 1:** Statistical description of continuous variables.

Variables	Minimum	First Quartile	Median	Mean	Third Quantile	Maximum
age	27	53	60.5	59.8	67	86
BMI (kg/m2)	14.2	20.7	23.8	24.4	26.8	46.5
WC (cm)	63	81	89	89.7	98	141
WHR	0.716	0.882	0.942	0.935	0.989	1.146
WHtR	0.373	0.494	0.548	0.554	0.607	0.865
SBP (mmHg)	93	120.8	136	138	153	258
DBP (mmHg)	54	73	84	84.1	91	167
FPG (mg/dl)	52	90	103	135.5	137	542
TC (mg/dl)	92	146	170.5	172.5	193	342
TG (mg/dl)	31	97	143	196.5	226	1407
HDL-C (mg/dl)	20	37	43.5	46.2	54.3	99

Note: BMI—body mass index; WC—waist circumference; WHR—waist-to-hip ratio; WHtR—waist-to-height ratio; SBP—systolic blood pressure; DBP—diastolic blood pressure; FPG—fasting plasma glucose; TC—total cholesterol; TG—triglyceride; HDL-C—high-density lipoprotein cholesterol.

**Table 2:** Gender-based difference analysis.

Variables	No. (%)	Male	Female	*p*-Value
No. (%)	280	217 (77%)	63 (23%)	
age		59.39	61.03	0.39
BMI		24.31	24.61	0.68
BMI category				0.69
Non-obesity	212 (76%)	166	46	
Obesity	68 (24%)	51	17	
WC		90.04	89.29	0.67
WC category				<0.001 ***
Non-obesity	129 (46%)	112	17	
Obesity	151 (54%)	105	46	
WHR		0.943	0.909	0.0013 **
WHR category				0.07
Non-obesity	86 (31%)	73	13	
Obesity	194 (69%)	144	50	
WHtR		0.547	0.576	0.014 *
WHtR category				0.09
Non-obesity	74 (26%)	63	11	
Obesity	206 (74%)	154	52	
SBP		137.48	139.65	0.52
DBP		84.44	82.98	0.50
Hypertension				0.97
Normal	145 (52%)	113	32	
Abnormal	135 (48%)	104	31	
FPG		138.51	125.08	0.27
Hyperglycemia				0.12
Normal	193 (69%)	144	49	
Abnormal	87 (31%)	73	14	
TC		168.12	187.59	0.0014 **
TG		196.59	196.27	0.99
Hyperlipidemia				0.09
Normal	187 (67%)	151	36	
Abnormal	93 (33%)	66	27	
HDL-C		44.27	52.73	<0.001 ***

Note: BMI—body mass index; WC—waist circumference; WHR—waist-to-hip ratio; WHtR—waist-to-height ratio; SBP—systolic blood pressure; DBP—diastolic blood pressure; FPG—fasting plasma glucose; TC—total cholesterol; TG—triglyceride; HDL-C—high-density lipoprotein cholesterol. *—significant; **—highly significant; ***—very highly significant.

**Table 3:** Adjusted odds ratios (ORs) for cardiovascular disease (CVD) markers for obesity-related indicators.

Obesity-Related Factors	Hypertension				Hyperglycemia				Hyperlipidemia			
	Unadjusted		Adjusted		Unadjusted		Adjusted		Unadjusted		Adjusted	
	OR (95%CI)	*p*	OR (95%CI)	*p*	OR (95%CI)	*p*	OR (95%CI)	*p*	OR (95%CI)	*p*	OR (95%CI)	*p*
BMI	1.07 (1.02–1.13)	0.005 **	1.10 (1.04–1.16)	<0.001 ***	1.03 (0.98–1.08)	0.29	1.04 (0.98–1.09)	0.17	1.12 (1.06–1.18)	<0.001 ***	1.11 (1.06–1.18)	<0.001 ***
WC	1.04 (1.02–1.06)	<0.001 ***	1.05 (1.02–1.07)	<0.001 ***	1.01 (0.99–1.04)	0.16	1.02 (0.99–1.04)	0.14	1.04 (1.02–1.07)	<0.001 ***	1.04 (1.02–1.07)	<0.001 ***
WHR	1.07 (1.03–1.11)	<0.001 ***	1.08 (1.04–1.12)	<0.001 ***	1.04 (1.00–1.08)	0.05	1.03 (0.99–1.07)	0.12	1.07 (1.03–1.12)	<0.001 ***	1.09 (1.05–1.14)	<0.001 ***
WHtR	1.06 (1.03–1.10)	<0.001 ***	1.06 (1.03–1.10)	<0.001 ***	1.02 (0.99–1.05)	0.20	1.02 (0.99–1.06)	0.14	1.08 (1.04–1.11)	<0.001 ***	1.07 (1.04–1.11)	<0.001 ***
BMI category	1.76 (1.02–3.08)	0.046 *	2.09 (1.18–3.77)	0.013 *	1.82 (1.02–3.20)	0.040 *	2.02 (1.12–3.64)	0.018 *	3.06 (1.74–5.41)	<0.001 ***	2.95 (1.67–5.25)	<0.001 ***
WC category	1.92 (1.19–3.10)	0.007 **	2.00 (1.22–3.31)	0.006 **	0.94 (0.57–1.56)	0.81	1.02 (0.61–1.73)	0.93	3.16 (1.87–5.47)	<0.001 ***	3.03 (1.77–5.31)	<0.001 ***
WHR category	2.74 (1.62–4.74)	<0.001 ***	2.74 (1.59–4.81)	<0.001 ***	1.06 (0.61–1.85)	0.84	1.09 (0.63–1.93)	0.75	3.53 (1.91–6.93)	<0.001 ***	3.53 (1.89–6.98)	<0.001 ***
WHtR category	5.49 (2.99–10.63)	<0.001 ***	5.38 (2.89–10.54)	<0.001 ***	1.43 (0.80–2.64)	0.24	1.46 (0.81–2.73)	0.22	4.97 (2.46–11.20)	<0.001 ***	5.18 (2.53–11.81)	<0.001 ***

Note: BMI—body mass index; WC—waist circumference; WHR—waist-to-hip ratio; WHtR—waist-to-height ratio; OR—odds ratio; CI—confidence interval. *—significant; **—highly significant; ***—very highly significant.
